# The Socioecological Model as a framework for exploring factors influencing childhood immunization uptake in Lagos state, Nigeria

**DOI:** 10.1186/s12889-021-10922-6

**Published:** 2021-05-05

**Authors:** Abisola Olaniyan, Chinwoke Isiguzo, Mary Hawk

**Affiliations:** Department of Behavioral and Community Health Sciences, University of Pittsburgh Graduate School of Public Health, 130 De Soto Street, 6120 Public Health, Pittsburgh, PA 15261 USA

**Keywords:** Immunization, Vaccination, Vaccines, Socioecological Model

## Abstract

**Background:**

Nigeria is one of the ten countries globally that account for 62% of under- and unvaccinated children worldwide. Despite several governmental and non-governmental agencies’ interventions, Nigeria has yet to achieve significant gains in childhood immunization coverage. This study identifies intrapersonal, interpersonal, organizational, community, and policy-level factors that influence childhood immunization uptake from various stakeholders’ perspectives using the Socioecological Model (SEM).

**Methods:**

Using the Socioecological Model as a guiding framework, we conducted ten focus group sessions with mothers/caregivers and community leaders residing in Lagos state and nine semi-structured interviews with healthcare workers who provide routine immunization services in Lagos state primary healthcare facilities. We performed a qualitative analysis of focus groups and semi-structured interviews using deductive coding methods.

**Results:**

The study sample included 44 mothers/caregivers and 24 community leaders residing in Lagos State, Nigeria, and 19 healthcare workers (routine immunization focal persons) working in the primary healthcare setting in Lagos state. Study participants discussed factors at each level of the SEM that influence childhood immunization uptake, including intrapersonal (caregivers’ immunization knowledge, caregivers’ welfare and love of child/ren), interpersonal (role of individual relationships and social networks), organizational (geographical and financial access to health facilities, health facilities attributes, staff coverage, and healthcare worker attributes), community (community outreaches and community resources), and policy-level (free immunization services and provision of child immunization cards). Several factors were intertwined, such as healthcare workers’ education of caregivers on immunization and caregivers’ knowledge of vaccination.

**Conclusions:**

The reciprocity of the findings across the Socioecological Model levels emphasizes the importance of developing multi-pronged interventions that operate at multiple levels of the SEM. Our results can inform the design of culturally appropriate and effective interventions to address Nigeria’s suboptimal immunization coverage.

## Background

As a cost-effective method of reducing morbidity and mortality resulting from vaccine-preventable diseases, vaccines are one of the greatest global health achievements [1]. Childhood immunization programs prevent two to three million deaths every year by decreasing the incidence of diseases such as diphtheria, pertussis, tetanus, measles, and tuberculosis [2]. They also protect the unimmunized by conferring herd immunity achieved with high rates of timely immunization [3]. From 2000 to 2016, global coverage rates for the childhood Diphtheria-tetanus-pertussis (DTP3) vaccine increased from 72 to 86% percent, while in the same years, childhood immunizations for measles increased from 72 to 85% [4]. Despite these improvements, a significant need remains; in 2016, approximately 20 million children did not receive the required three-dose regimen of DTP3, and more than 21 million children missed their first measles vaccine [4].

Nigeria accounts for a significant percentage of children that didn’t receive or missed these vaccines, accounting for 3 million of the 20 million under- and unvaccinated children in the world [5]. According to the Multiple Indicator Cluster Survey, in 2017, Nigeria achieved full immunization coverage of only 23% of its children by their first birthday [6].

One of the responsibilities of the Nigerian National and State Primary Health Care Development Agencies is to develop and implement policies and programs to improve childhood immunization services. These interventions aim to overcome specific supply- and demand-side barriers related to affordability, accessibility, availability, awareness, and knowledge of immunization services. Despite these interventions from the government and non-governmental partners, such as the World Health Organization (WHO), UNICEF, and the Clinton Health Access Initiative (CHAI), Nigeria has yet to reach the proportion of children covered by all vaccines that is needed to support the attainment of the United Nation’s global Sustainable Development Goal (SDG).

Multiple studies have examined factors influencing childhood immunization uptake in Nigeria to guide the development of programs and policies to improve childhood immunization coverage. These studies have focused mainly on individual-level factors, including maternal education and religious or cultural beliefs, placing the burden of interventions on the individual [7–9]. However, the Socioecological Model (SEM) demonstrates that individual behavior is shaped by factors at multiple levels [10–12]. The underlying premises of the SEM are that individual behaviors both influence and are influenced by multilevel factors and the social environment and that understanding influences within these multiple levels is necessary to resolve and prevent public health problems [12].

The SEM describes how individual, interpersonal, organizational, community, and policy factors shape population health [12]. The intrapersonal level of the SEM represents individuals’ characteristics, including knowledge, attitudes, and behaviors [11]. These are consistent with concepts posited by individually-focused behavioral theories, such as the Health Belief Model, that are expected to affect childhood immunization uptake or practice [13, 14]. The interpersonal level of the SEM describes individuals’ familial and social networks that may influence healthcare practices and contribute to various experiences [11]. Strong interpersonal dynamics in these relationships are thought to significantly affect an individual’s physical and mental health and health decision-making [15–20]. Studies have shown that social influence from interpersonal relationships significantly affects health behaviors, including health-seeking behavior, breastfeeding practices, and uptake of family planning methods [21–26]. The institutional level describes the roles that characteristics and operations of social institutions, including health facilities and their health workers, play in shaping health care decision-making [11]. Community-level determinants include basic resources and the social and physical environment that comprise the greater community [11]. The nature of infectious diseases, including those that are vaccine-preventable, highlights the importance of the social context of risk perception on vaccine uptake since one’s concerns about their children becoming infected or transmitting disease to others would likely impact vaccine decision-making. Additionally, herd immunity plays a vital role in the spread of infection, making immunization a community-based effort [3]. These factors may influence the actions of community leaders and community-based health workers. Social dynamics within a community, therefore, play a role in the perception of risk. The outermost tier of the SEM, policy, accounts for the local, state, and national laws and policies that impact health practices [11]. Policies play a fundamental role in access to healthcare services, utilization of healthcare services, and the adoption of healthy behaviors [27]. For example, there is ample evidence that universal healthcare coverage increases access to healthcare services, including immunization [27–29].

Although the SEM is widely accepted as a framework for understanding health determinants, most research studies only partially explore determinants nested within the SEM and do not consider the model’s entirety when examining factors related to immunization uptake and intervention development. Vaccination interventions in Nigeria have focused significantly on supply-side challenges with inadequate investigation into relevant demand-side needs. Without a full understanding of distal and proximal determinants of childhood immunization uptake, it is unlikely Nigeria will be able to create and sustain the policy and intervention initiative needed to meet and maintain SDG goal.

To fill this knowledge gap, our study used the socioecological model as a conceptual and organizing framework to explore factors influencing childhood immunization from stakeholders’ perspectives, including mothers and other caregivers, community leaders, and healthcare providers in Lagos state, Nigeria. Investigating these socioecological influences on childhood immunization practices from lived perspectives is critical to the development of comprehensive and effective interventions.

## Methods

### Study design

The study team employed a qualitative descriptive approach using both focus group sessions and semi-structured interviews. We followed the Consolidated Criteria for Reporting Qualitative Research (COREQ) guidelines [30]. To explore our research question, we developed separate interview guides for focus groups and interviews, but both were based on a review of extant literature and the SEM, which conceptually informed both interview guides. The interview guide for the focus group session with mothers/caregivers and community leaders was available in Yoruba, the local language, and English. For the purpose of this study, we defined caregivers as mothers and other adults charged with supervising children. Henceforth, we will refer to mothers and caregivers as simply “caregivers” for ease of reference. We used convenience sampling to recruit study participants. Caregivers and community leaders were recruited from across all the 20 Local Government Areas (LGAs) in Lagos state. They were approached face-to-face through multiple avenues including outreach at regular immunization clinics, postnatal clinics, and within the community. Immunization officials from the Lagos State Primary Health Care Board assisted with recruiting health care workers and community leaders. The community leaders and health care workers assisted with recruitment of the mothers/caregivers and other community members. Potential participants were informed about the opportunities to take part in interviews or focus groups. We aimed to recruit at least two caregivers, one community leader and one healthcare worker from each of the 20 LGAs in Lagos state.

Eligibility criteria for mothers/caregivers and community leaders were age ≥ 18 years, living in Lagos state, and understanding and speaking either English or Yoruba. Healthcare workers were eligible if they provided routine immunization at health facilities in Lagos state and understood and spoke English or Yoruba. We informed study participants of the voluntary nature of the study and collected verbal consent prior to data collection. All caregivers and community leaders that were approached agreed and consented to participate in the study, and there were no drop outs in focus groups. All recruited health workers agreed to participate in the study but one health worker had to drop out of the semi-structured interview. We had focus group sessions with caregivers and community leaders from across all 20 LGAs in the state and semi-structured interviews with healthcare workers from 19 of the 20 LGAs in Lagos state. All interviews and focus groups were conducted by the first author (AO) who is female and has training and experience in the conduct of qualitative research. The interviewer met with all participants prior to the commencement of study data collection to provide them with basic information about the interview and the study aims. Participants were also informed that the researcher was a physician and PhD student with an interest in childhood immunization, as well as an indigene of the state.

The interviewer conducted ten focus group sessions with caregivers and community leaders (*n* = 68) and nine semi-structured interviews with healthcare workers (19 routine immunization focal persons). Each focus group session had 6–8 participants. Focus group participants were grouped based on the proximity of their LGA of residence. Most of the semi-structured interviews were conducted with more than one healthcare worker. Interviews and focus groups took place in person at various study sites. The study sites included the Local Government Immunization Officers (LIO) office in the LGA Headquarters and public primary health facilities selected by the study participants. Eight of the ten focus group discussions were held in public primary facilities while two were held in the LIO’s office. Seven of the nine semi-structured interviews were held in the public primary facilities while the remaining two were held in the LIO’s office. The interviewer conducted the interviews and focus groups in English or Yoruba, each ranged between 30 and 90 min in length, and were audio-recorded and transcribed verbatim, including those conducted in Yoruba. Focus groups and interviews conducted in Yoruba were translated into English for analysis. To ensure accurate translation of data collected in Yoruba, a member of our research team who speaks Yoruba fluently reviewed the transcripts. The interviewer made field notes during the focus groups and interviews to allow for reflexivity. Focus groups and interviews were conducted until saturation was achieved. All qualitative data were de-identified prior to analysis. The Health Research and Ethics Committee of the Lagos State University Teaching Hospital approved the study protocol. The study protocol was deemed exempt from review by the University of Pittsburgh Institutional Review Board. We also received a social approval from the Lagos State Primary Health Care Board (LSPHCB).

### Data analysis

All transcripts were imported into NVivo 12 for coding and analysis. We used an iterative process of thematic analysis employing a deductive coding approach based on the SEM framework while also exploring for emerging themes. To build the initial codebook, two study team members read four of the transcripts and developed a set of codes grounded in our research question and the SEM. We used an iterative process of group discussion and recoding to develop a codebook collaboratively. Members of the study team discussed and refined codes until they were fully understood and agreed upon by all. Two team members separately coded two of the interviews and then compared results, again refining codes and code application, and comparing coding structures until there was full agreement. Three team members coded the rest of the transcripts, double-coding 8 of the 19 transcripts to ensure they applied codes consistently. As team members identified new themes, we discussed them, reviewed relevant field notes, and added them to the codebook once we reached an agreement on the theme. The team held regular meetings to compare and contrast similarities and differences among the researchers’ coding. In addition, we revisited the transcripts’ texts several times to ensure we captured the essence of the meaning, specifically focusing on cultural nuances and complex ideas or themes. The study team members compiled and reviewed excerpts from the coded transcripts, then discussed and analyzed emerging and prominent themes. All identified themes were able to be grouped into an SEM level with no emerging themes outside of the SEM framework. Themes are presented according to the levels of the SEM and augmented with illustrative quotes.

## Results

### Demographic characteristics

From across all 20 LGAs in Lagos state, we recruited 87 study participants. Participants included 44 caregivers, 24 community leaders and 19 nurses who were routine immunization focal persons in public primary health facilities. Most of the study participants were female (77/87). All caregivers [31] and nurses [19] were females. Ten of the 24 community leaders were male and 14 were female.

**Fig. 1:**
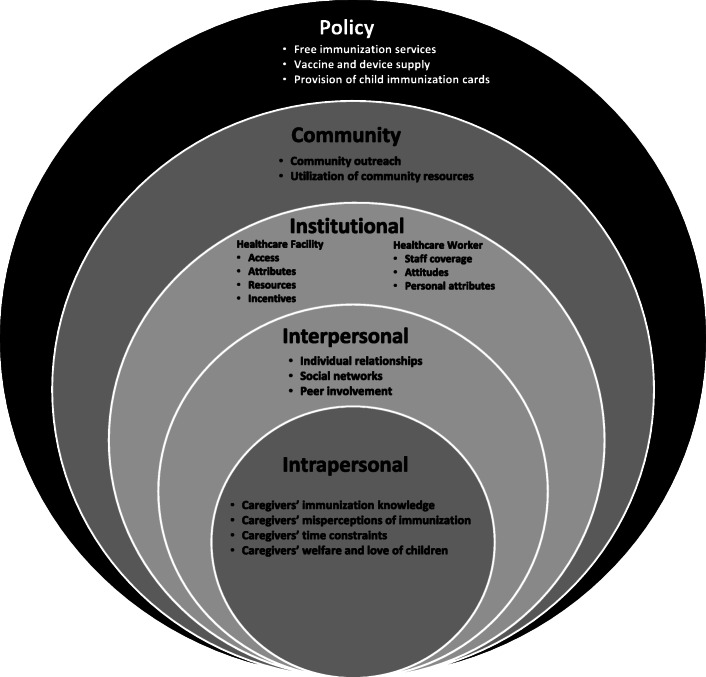
SEM Categories and Identified Concepts

We grouped the concepts identified in our analysis into five thematic categories in accordance with the SEM framework, including (a) intrapersonal (b) interpersonal (c) institutional (d) community and (e) policy (Fig. 1). Despite the differentiation of the five categories, many variables were intertwined, which is consistent with the concept of reciprocity described in the SEM.

### Intrapersonal-level factors

Participants in our study shared several individual-level factors that influenced caregivers’ uptake of childhood immunization services. These intrapersonal factors included those that are intrinsic, such as knowledge, and extrinsic, referring to factors such as the caregivers’ time constraint. Participants reported caregivers’ accurate and adequate knowledge of immunization services as being a positive influence on immunization uptake. Essential elements of knowledge included understanding vaccine benefits, such as preventing disease, being aware of the immunization schedule delineating the age at which specific vaccines were recommended, and the potential adverse events following immunization. A related but separate theme was caregivers’ negative beliefs and misperceptions of immunization.

One of the most frequently discussed individual-level factors was caregivers’ time constraints or conflicting work schedules, which they thought negatively affected immunization uptake. This theme was related to an organizational-level theme discussed below: clinic waiting times. Caregivers’ welfare and love of children was another individual-level factor that participants described as being important to consider for its role in immunization as it could facilitate uptake for people who were aware of the importance of immunization or act as a barrier for those who had misperceptions about immunization. This theme relates to the affection or well-being of their child that drives vaccine decisions.

The following excerpts capture discussions about the importance of individual-level determinants of vaccine uptake.

*“I know someone that stopped coming because she said she couldn’t be wasting all her time in the health center that she has to go to the market.” [Female Caregiver]*

*“P1: To buttress all she has said, it is true when the baby is immunized that they might run temperature. If you check my baby’s temperature, he is still running temperature. So, if he is running temperature, parents will feel concerned and wonder maybe it is the injection administered. I might decide not to come again because if he wasn’t immunized, he will not run temperature. If you check my card, you will observe that I have missed the injections many times.*

*I: Why did you miss them?*

*P1: He was running temperature, I thought it wasn’t necessary to immunize a baby when he is running temperature I didn’t ask and didn’t take him for immunization until yesterday before I took him for immunization.” [Female Caregiver]*

Individual-level factors reported by participants were affected by determinants across all other SEM levels, especially those relating to members of caregivers’ families or social networks.

### Interpersonal-level factors

In our study, participants discussed interpersonal factors, including those relating to the caregivers’ relationships and social networks, including family, friends, and neighbors. Some caregivers in our focus groups discussed how other mothers within their networks had educated them about immunization and encouraged them to immunize their wards. They emphasized the importance of peers sharing their knowledge of vaccination with their peers. Caregivers also highlighted the roles that husbands, mothers, and mothers-in-law played in their decision-making about childhood immunization. Intergenerational roles were seen as being particularly influential on immunization decision-making; however, the impact of these roles varied based on the level of knowledge of these influencers.

*“I learnt about immunization from my mom because my mother told me that she has never missed any of her immunization and she taught me the importance of it. I don’t take my children to the hospital because they don’t get sick because I make sure I complete their immunization. She even told me to the extent that then they always give them a card when any child completes his/her immunization they will give you a card so everybody has that card meaning the child completed immunization. That card meant a lot to everybody and will encourage mothers to complete immunization.” [Female Caregiver]*

However, we also found that intergenerational influences could also serve as a barrier to vaccine uptake in situations where senior family members carried misinformation or negative attitudes about immunization.

*“P3: The reason why I didn’t immunize my baby is because my mother-in-law is in the village.*

*I: Okay!*

*P3: My mother-in-law said that they don’t allow it … .It had happened to her before. It eventually killed the child. So, that is the reason why she had refused me. But I know things have changed. That was during her era. She was adamant that she wouldn’t allow me leave her sight until the baby is six months old. That … … . it is a must because things have changed; the man might decide to bring in another woman if there is no woman around. I pleaded with her till Monday before I was able to bring the child on Tuesday.” [Female Caregiver]*

### Institutional-level factors

Our study participants discussed health facilities and healthcare workers’ impact on childhood immunization decision-making and uptake in Lagos. Some important facility-related factors included access, either geographical, such as bad roads leading to the facility, or financial, including transportation costs, which negatively impacted immunization uptake. However, the attributes of health facilities were also discussed. Facilities with poor waiting areas or lack of adequate ventilation or cooling systems such as fans or air conditioners made some caregivers reluctant to access them. Resources offered by facilities were also influential, and participants highly regarded facilities that provided free consumables such as cotton wool, gloves, and drugs. The mothers also valued incentives offered by facilities, and the incentives described included mosquito nets or baby products for new mothers.

Roles, attitudes, and personal attributes of health workers were also described in much detail. Understaffed facilities resulted in long wait times, so caregivers placed value on facilities with adequate staff coverage, including record-keeping personnel. However, there were also many discussions about attitudes and personal attributes such as cleanliness of the healthcare workers as influencing the likelihood of accessing childhood immunization. Some participants had previously had experiences with healthcare workers they described as rude, which they reported would negatively affect vaccine access in the future. Additionally, some described healthcare workers who looked untidy as a potential deterrent to immunization uptake by caregivers. Others discussed the importance of having healthcare workers who could educate them about the importance of immunizations for their children as a positive contributor to improving caregivers’ immunization acceptance.

Participants described facility- and health worker-related factors that influence childhood immunization uptake as follows:

*“I: What are the other things that make you reluctant to go back for the immunization or to even start it?*

*R: The insults from health care workers” [Female Caregiver]*

*"For example, this place wasn't the way it looks now, but when the place was equipped, and there are doctors on the ground. Since all had been put in place, people have been trooping in. Especially if you come around on Mondays, you will see the turnout of people. This gives people the confidence that when they come, they will be attended to."[Male Community Leader]*

*"We spend almost the whole day in the health center to immunize our children. If there are more nurses, we will be able to get immunization quickly and do other things that day." [Female Caregiver]*

### Community-level factors

In our study, themes that emerged as community-level influences on immunization update encompassed factors within the environments where people live, including influences of informal networks such as community leaders and the availability of community resources. Participants discussed the positive impact of a community outreach, which they described as immunization services provided by healthcare workers within the community to increase immunization coverage, especially in underserved areas. Participants also described the positive value of house-to-house immunization campaigns conducted by trained volunteers, such as National Immunization Plus Days (NIPDs), during which supplemental oral polio vaccine is distributed on a house-to-house basis to children less than five years of age.

Furthermore, caregivers, community leaders, and healthcare workers in our study described the importance of leveraging community resources such as community leaders, including traditional and religious leaders, and those in formal community leadership positions such as members of the Ward Development Committee to encourage caregivers’ immunization uptake. One of the participants described the effect of community influences on childhood immunization uptake as follows:

*"Like we have outreaches now that we do every Wednesday. I think the outreach is helping us a lot. The mothers that can't come to PHC, the outreach workers- the people that go for outreaches go to their communities to immunize them, and that is even increasing our immunization coverage."[Female Healthcare Worker]*

### Policy-level factors

In Nigeria, various laws and policies govern immunization services, which differ between federal, state, and local governments. Participants in our study described the critical importance of free immunization services, a national health policy meant to provide these free services in all health facilities, which enables childhood immunization uptake. However, participants discussed how the availability of free immunization services differed between public or private health facilities.

*I: What are the programs or policies or things that government has done that encourages you to immunize your child.*

*R: It is free. But I think that it is why some health centers are usually very full and then we have to wait for a very long time to get immunization for our children. [Female Caregiver]*

Another policy described by participants as a positive influence to vaccine uptake was the provision of child immunization cards, a national policy whereby all children are provided with an immunization card to record vaccines taken, and subsequent appointment dates. Participants in our study indicated that the child immunization cards helped caregivers to track immunization schedules and helped caregivers to access vaccines in different healthcare facilities if they were traveling. This is because the immunization card provides a record of the vaccines the child has received and those due for subsequent visits. Participants also discussed some vaccines such as Rotavirus, which are captured in the child immunization card but not offered by public health facilities, although they are available in some private facilities at a cost.

“*I: What are the things that you think the government still need to do?*

*R: Like this “Rota-virus” that is in the card they haven’t started giving us … .. I think I heard it’s supposed to be free that “Rota-virus”. Is it supposed to be free?” [Female Caregiver]*

## Discussion

Some previous studies have applied the SEM to explore childhood immunization factors for certain vaccines, such as the immunization for tuberculosis [32]. However, to our knowledge, no study has explored Nigerian childhood immunization uptake for all vaccines guided by the SEM from the perspective of various immunization stakeholders, including mothers/caregivers, community leaders, and healthcare workers. Our study shows that factors at all SEM levels affect childhood immunization uptake and decision-making by caregivers. The SEM is based on the concept that individuals are embedded within social networks, which are in turn embedded within institutions and communities, and that all of these are impacted by policies that directly or indirectly influence health decision-making and practices [11, 12]. Though previous work examining childhood immunization uptake in Nigeria has explored individual-level determinants, such as accuracy of vaccine information and availability [8, 9, 33], our qualitative study demonstrates that these immunization behaviors are shaped by distal factors, including social network, community, organizational, and policy influences.

Our findings corroborate other studies that have reported that people who believe that individuals within their social network want their children to be vaccinated are more likely to accept vaccines [32, 34–36]. Additionally, studies have also shown that as acceptance of vaccination within a community increases, making it a social norm, vaccine uptake by people within that community will also increase [34, 37]. At the institutional level, literature has shown that availability and access to health care facilities significantly affect care-seeking behavior and utilization of health services [38–41]. In addition, patient-health care worker communication significantly impacts healthcare utilization, decision-making, acceptance of recommended health behaviors, and medical management [42–44]. Researchers have demonstrated that immunization uptake increases when healthcare workers educate and recommend immunization to caregivers [31, 39]. These reports are similar to findings in our study.

Our study findings at the community level of the SEM also complement the results of numerous studies that have highlighted multiple factors impacting vaccine uptake at this level, including the incidence and prevalence of the disease in one’s community. Additionally, in some communities, especially those in developing countries, community and traditional leaders have played a role in their communities’ immunization practices [32, 39, 45]. At the policy level, Lagos state has an immunization supply chain policy, ensuring the availability of adequate vaccines and devices at health facilities. In addition, the country has various policies in place which participants discussed as positive influencers of immunization uptake including free immunization services and the provision of child immunization cards. Some vaccines in the Nigerian National Immunization Schedule such as Rotavirus vaccine and Human Papilloma Virus vaccine are yet to be introduced by the country into its immunization program, and therefore are not available in the public health facilitiesthat provide free immunization services. Mothers who would like to provide these vaccines for their wards have to get them from private facilities for a fee.

Our study complements findings from other immunization studies in Nigeria and other countries exploring factors influencing immunization uptake at one or more Socioecological Model levels [9, 32, 33, 39, 40]. The factors at the different SEM levels are not standalone; they influence each other over time. In our study, we found that organizational efforts in which health workers provided immunization health talks to caregivers as early as during antenatal care coupled with community efforts in which local leaders create awareness about the relevance of immunization both influence mothers’ knowledge of immunization benefits and assist with debunking misperceptions. Additionally, policies providing free immunization services affect financial access to health facility services, thereby influencing caregivers’ immunization decisions for their wards. Furthermore, organizational level factors such as the staff numbers, healthcare workers’ roles, and healthcare workers’ attitudes impact the caregivers’ clinic wait times and influence the caregivers’ vaccine decisions. For example, the provision of free immunization services in the absence of awareness creation among caregivers and caregivers’ knowledge of immunization benefits will not foster immunization uptake. Therefore, the most effective immunization service requires a combination of factors across multiple SEM levels.

Our study is not without limitations. First, the use of a convenience sample creates the threat of selection bias. Second, we recruited all participants from Lagos state; thus, we cannot compare our findings with other states that might have different policies and programs in place. Third, one of the researchers conducting this study has prior experience with immunization programming in Lagos state which could bias the interpretation of results. To foster reflexivity and mitigate this bias, we used multiple coders, including those without prior experience with immunization in Lagos state, Nigeria.

Despite these limitations, this research provides a unique and insightful point for understanding factors influencing childhood immunization decision-making from all stakeholders’ perspectives (mothers/caregivers, community leaders, and healthcare workers). This study is the first attempt at exploring childhood immunization uptake guided by SEM from these stakeholders’ perspectives.

## Conclusions

This study demonstrates elements of the SEM that influence childhood immunization uptake; however, additional research is needed to elucidate barriers and facilitators affecting caregivers’ decisions to immunize their children. Our stakeholder-driven findings have important implications for both policy and practice as they provide a framework to guide intervention planning, development, and implementation for government and partners tasked with improving immunization outcomes. A comprehensive understanding of the influence of the different levels of the SEM on caregivers’ decision-making for childhood immunization can inform intervention planning. Our findings should encourage all immunization stakeholders, including government officials and partner agencies, to design interventions that simultaneously target multiple SEM levels to foster positive immunization decision-making and increase childhood immunization uptake. The findings have the potential for an even greater impact since lessons learned regarding childhood immunization decision-making may be applied to other vaccine decisions. Given the COVID-19 pandemic and the availability of new COVID-19 vaccines, it will be important to apply SEM to immunization uptake challenges to ensure coverage needed to change the course of morbidity and mortality rates from coronavirus infection. Additionally, implementing these findings will facilitate prompt uptake of new vaccines such as the Human Papilloma Virus vaccine scheduled for introduction into the routine immunization schedule of the country. It is critical that future public health interventions build on stakeholder input and consider all aspects of the SEM in order to achieve SDG and reduce childhood death due to vaccine-preventable disease.

## Data Availability

Data sharing is not applicable to this as no datasets were generated or analyzed during the current set. All data collected were qualitative in nature.

## Abbreviations

CHAI: Clinton Health Access Initiative
DTP3: Third dose of the diphtheria-tetanus-pertussis vaccine
LSPHCB: Lagos State Primary Health Care Board
NIPDs: National Immunization Plus Days
PHC: Primary Health Centre
SDG: Sustainable Development Goal
SEM: Socioecological Model
UNICEF: United Nations Children’s Fund
WHO: World Health Organization
